# Increased Hepcidin Levels During a Period of High Training Load Do Not Alter Iron Status in Male Elite Junior Rowers

**DOI:** 10.3389/fphys.2019.01577

**Published:** 2020-01-21

**Authors:** Martina Zügel, Gunnar Treff, Jürgen M. Steinacker, Benjamin Mayer, Kay Winkert, Uwe Schumann

**Affiliations:** ^1^Department of Internal Medicine, Division of Sports and Rehabilitation Medicine, Ulm University, Ulm, Germany; ^2^Institute of Epidemiology and Medical Biometry, Ulm University, Ulm, Germany

**Keywords:** ferritin, transferrin, soluble transferrin receptor, rowing, exercise, inflammation, iron supplementation

## Abstract

The liver-derived hormone hepcidin plays a key role in iron metabolism by mediating the degradation of the iron export protein ferroportin 1 (FPN1). Circulating levels of hepcidin and the iron storage protein ferritin are elevated during the recovery period after acute endurance exercise, which can be interpreted as an acute phase reaction to intense exercise with far-reaching consequences for iron metabolism and homeostasis. Since absolute and functional iron deficiency (ID) potentially lead to a loss of performance and well-being, it is surprising that the cumulative effects of training stress on hepcidin levels and its interplay with cellular iron availability are not well described. Therefore, the aim of this study was to determine serum levels of hepcidin at six time points during a 4-week training camp of junior world elite rowers preparing for the world championships and to relate the alterations in training load to overall iron status determined by serum ferritin, transferrin, iron, and soluble transferrin receptor (sTfR). Serum hepcidin levels increased significantly (*p* = 0.02) during the initial increase in training load (23.24 ± 2.43 ng/ml) at day 7 compared to the start of training camp (11.47 ± 3.92 ng/ml) and turned back on day 13 (09.51 ± 3.59 ng/ml) already, meeting well the entrance level of hepcidin at day 0. Serum ferritin was significantly higher at day 7 compared to all other timepoints with exception of the subsequent time point at day 13 reflecting well the time course pattern of hepcidin. Non-significant changes between training phases were found for serum iron, transferrin, and sTfR levels as well as for transferrin saturation, and ferritin-index (sTfR/log ferritin). Our findings indicate that hepcidin as well as ferritin, both representing acute phase proteins, are sensitive to initial increases in training load. Erythropoiesis was unaffected by iron compartmentalization through hepcidin. We conclude that hepcidin is sensitive to rigorous changes in training load in junior world elite rowers without causing short-term alterations in functional iron homeostasis.

## Introduction

Iron deficiency (ID) with serum ferritin <20–30 μg/l (Di Santolo et al., 2008; Reinke et al., 2012; Auersperger et al., 2013; Ziemann et al., 2013; Peeling et al., 2014) is a frequently discussed phenomenon in endurance athletes with potentially deleterious effects on endurance performance due to reduced blood gas transport and cellular oxidative capacity (Haas and Brownlie, 2001; Dellavalle and Haas, 2012; Hinton, 2014; Stugiewicz et al., 2016). In healthy male endurance athletes, ID can occur due to increased loss of iron through sweat (Brune et al., 1986; DeRuisseau et al., 2002), hematuria (Siegel et al., 1979; McInnis et al., 1998), mechanical hemolysis (Telford et al., 2003), gastrointestinal bleeding (Stewart et al., 1984), insufficient nutritional uptake of iron, or combinations of these factors.

However, even if nutritional uptake of iron is sufficient, the capacity of enterocytes in the small intestine to absorb dietary iron under steady-state conditions (1–2 mg per day) is limited and most of the body iron comes from macrophages (20–25 mg per day) of the mononuclear phagocyte system, which internally recycles iron from senescent erythrocytes (Hentze et al., 2010). The monomeric glycoprotein transferrin transports iron in the bloodstream and binds to transferrin receptors (TfR) on target cells. Iron is released intracellularly after endocytosis of the receptor complex and is either incorporated into iron-containing proteins or stored as ferritin. Expression of the TfR starts to increase when iron stores are depleted or iron turnover is increased, e.g., during stimulated erythropoiesis (Beguin, 2003). Repeated or chronic inflammation – e.g., due to repeated exercise – and increased rates of erythropoiesis can lead to functional ID, where iron stores are adequate but iron transfer to the site of erythroblast production is insufficient due to compartmentalization of iron in the mononuclear-phagocyte system (Thomas et al., 2013). Notably, Reinke et al. (2012) reported that 70% of senior elite rowers, including participants of world championships, and professional soccer players were diagnosed with functional ID at the end of a competitive season, based on the clinical definition of functional ID (ferritin 30–99 μg/l or 100–299 μg/l and transferrin saturation <20%). However, in athletic populations, a serum ferritin cut-off of 30 μg/l has been suggested (Clénin et al., 2015). Furthermore, consensus exists that the diagnosis of functional ID should include serum ferritin levels as well as transferrin saturation (Anker et al., 2009; Eisenga et al., 2016). Circulating concentrations of the soluble transferrin recepetor (sTfR) and the ratio of sTfR to serum ferritin (sTfR-F index) have also been used as additional, reliable, and sensitive indicators for diagnosing functional ID (Malczewska et al., 2000, 2004; Schumacher et al., 2002a, c; Nikolaidis et al., 2003).

A key regulator of systemic iron homeostasis is the small, disulfide-rich hepatic bactericidal protein hepcidin, which acts by binding to the iron transporter ferroportin 1 (FPN1), causing the transporter to be internalized and degraded (Song et al., 2013). Hepcidin thereby prevents the export of iron from enterocytes, macrophages, and hepatocytes which results in the reduction of systemic iron availability. Hepcidin levels are suppressed when erythropoiesis is stimulated, such as during acute blood loss (Pasricha et al., 2016) and chronic endurance exercise in healthy, non-anemic, iron-sufficient men (Moretti et al., 2018), while it is increased in response to acute endurance exercise, iron overload and persistent, low-grade, sterile inflammation (Nicolas et al., 2002; Peeling et al., 2009, 2014; Newlin et al., 2012; Sim et al., 2012; Badenhorst et al., 2014, 2016; Diaz et al., 2015). The magnitude of the hepcidin response to acute endurance exercise appears to be dependent on baseline serum iron and ferritin levels (Peeling et al., 2014, 2017; Burden et al., 2015). Peeling et al. (2014) found that post-exercise hepcidin concentrations increased significantly solely in individuals with baseline serum ferritin levels >30 μg/l.

Most studies performed so far have shown that hepcidin levels return to baseline within 6 h after an acute bout of endurance exercise (Newlin et al., 2012; Sim et al., 2012; Diaz et al., 2015; Skarpanska-Stejnborn et al., 2015). However, there is a lack of data on the cumulative effects of regularly performed exercise training on the expression of iron regulatory factors and its implications for iron status and performance. We therefore aimed to study the effects of a 4-week endurance training on iron metabolism analyzing serum levels of hepcidin, ferritin, transferrin, iron, soluble transferrin receptor (sTfR), the calculated ferritin-index (sTfR/log ferritin), and transferrin saturation in male athletes with confirmed serum ferritin levels >30 μg/l. The reason for selecting a group of male junior world elite rowers is based on their high absolute total hemoglobin mass (Treff et al., 2014) and the fact that they undergo a training regime that is associated with a heavy metabolic load, especially during training camps (Steinacker et al., 2000). We hypothesized, based on findings by Ishibashi et al. (2017) in female runners, that serum hepcidin levels are elevated in periods consisting of high training loads, compared to lower load phases, possibly affecting the iron status of high performance athletes in an unfavorable manner.

## Materials and Methods

### Study Participants and Experimental Protocol

**FIGURE 1:**
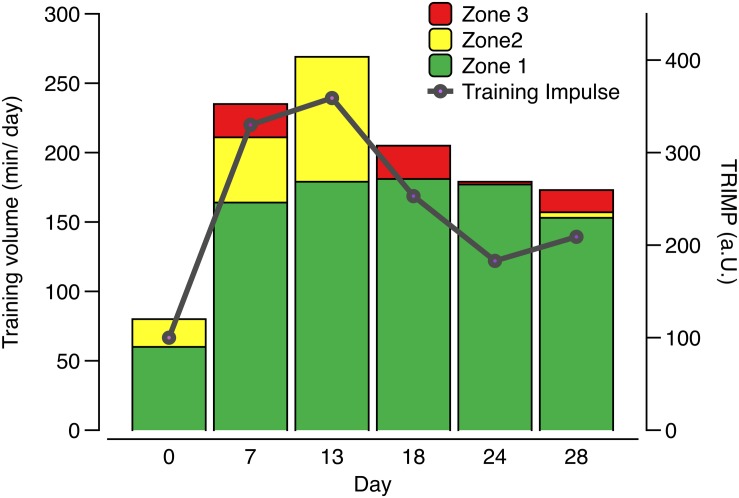
Average daily training volume, training intensity distribution, and resulting training load defined as TRIMP (intensity zone × duration) during 4 days prior to each testing time point in male junior world elite rowers during a 4-week pre-competition training camp before the junior world championships. Zone 1 refers to intensities of 50–70% of maximal heart rate, zone 2 to 70–80%, and zone 3 to 80–100%.

The ethical review board of the University of Ulm approved this study (121-09) which complied with the Declaration of Helsinki. All participants gave written informed consent and parental consent was obtained for athletes <18 years of age. In total, eight male junior world elite rowers of the German junior national team participated in the study (Table 1). They were tested at six time points (day 0, 7, 13, 18, 24, and 28) during a 4-week training camp in preparation for the junior world championships, where training intensity as well as training volume and therefore total training load were elevated at multiple timepoints compared to the usual training during the rest of the year. The cumulated training volume of 1 week (7 days) prior to the respective testing time points (0, 7, 13, 18, 24, and 28 days) was 780 min/week or 104 km/week (time point day 0); 1700 min/week or 180 km/week (time point day 7); 1435 min/week or 202 km/week (time point day 13); 1240 min/week or 170 km/week (time point day 18), and 840 min/week or 133 km/week (day 24 and day 28). Since training intensity distribution changed throughout the training camp, we calculated the overall training load using an exercise score (TRIMP-model), where training load is a product of training time and intensity zone, the latter being defined by heart rate zones of 50–70% (zone 1), 70–80% (zone 2), and 80–100% (zone 3) (Foster et al., 2001). For example, 50 min training spent in zone 1 and 10 min spent in zone 3 will result in a training load or TRIMP of 50×1 + 10×3 = 80 a.U. As shown in Figure 1, training load was low at baseline (day 0), high before days 7 and 13 and moderate before days 18, 24, and 28 due to the recovery phase to prepare for the competition. To better reflect the direct impact of the previous training days on the main outcome variables, we averaged the training data of 4 days prior to each timepoint to calculate the TRIMP.

**TABLE 1:** Anthropometric, endurance and performance data of *N* = 8 male junior world elite rowers.

**Variable**	**Value**
Age, years	17.80.4
Standing height, cm	1914
Body mass, kg	84.48.3
Body fat,%	9.44.2
BMI, kg/m^2^	23.11.6
Power 4 mmol/l [blood lactate], W	35116
V̇O_2_max, l/min	5.40.2
V̇O_2_max, ml/min/kg	633
Placing WCh	1 (1–3)

*Data are arithmetic means ± standard deviations, placing at Junior World Championships (WCh) is given as median (minimum – maximum), where six of eight rowers won gold medals, one won silver, and one bronze.*

### Biological Measurements

Alterations of iron metabolism (hepcidin, iron, ferritin, transferrin, and sTfR) were analyzed in venous serum samples. Blood samples were drawn after an overnight fast (starting 08:00 pm until 06:00 am) from the antecubital vein in the seated position ≥14 h after the last training session in the morning before breakfast. No rower suffered from anemia (hemoglobin [Hb] <120 g/l), and no athlete was classified as iron-deficient (serum ferritin levels <30 μg/l) (Clénin et al., 2015). Hemoglobin levels were measured via photometric testing with XE-2100, Sysmex. Ferritin and sTfR were measured by immunoturbidimetric testing (Cobas 6000, Roche). Transferrin was measured through immunoturbidimetric testing (Cobas 8000, Roche), and serum iron was determined by photometric testing (Cobas 6000, Roche). Total hepcidin was analyzed in serum samples using a commercial ELISA (USCN cat.-nr. 979hu). The ferritin index was calculated by dividing serum levels of the sTfR by log ferritin (sTFR/log ferritin). The transferrin saturation was calculated using the following formula: Tf-sat [%] = serum-iron [μg/dl]/serum-transferrin [mg/dl] × 70.9. To estimate changes in plasma volume, hematocrit was measured in capillary samples taken from the hyperaemized earlobe via centrifugation for 10 min at 10,000 rpm (Laborfuge A, Heraeus, Buckinghamshire, United Kingdom).

### Statistics

All data are expressed as arithmetic means ± standard deviation, unless otherwise specified. To analyze differences between days of measurement, a mixed linear model was applied to the data (PROC MIXED in SAS analytics pro V.9.4), using day of measurement as a fixed effect. Statistical significance was established as *p* < 0.05. In case of significant differences, a pairwise comparison between timepoints was calculated using Tukey’s test to adjust for multiple testing.

## Results

### Increased Serum Hepcidin Expression in Response to the Initial Increase in Training Load During a 4-Week Pre-competition Training Camp in Male Junior World Elite Rowers

**FIGURE 2:**
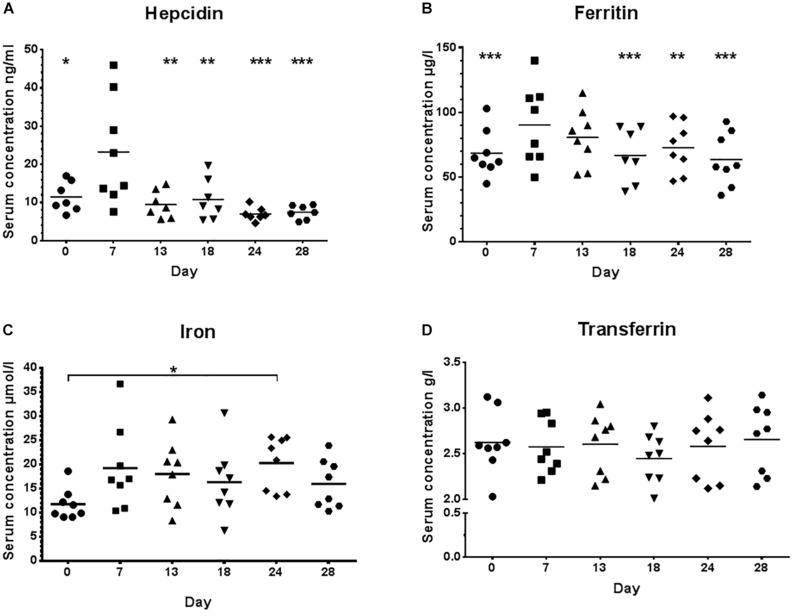
Serum hepcidin **(A)**, ferritin **(B)**, iron **(C)**, and transferrin **(D)** concentrations during low training load (day 0), high training load (day 7 and day 13), and moderate load before day 18, 24, and 28; ^∗∗∗^*p* < 0.001; ^∗∗^*p* < 0.01 and ^∗^*p* < 0.05 vs. day 7.

Serum hepcidin levels were significantly (*p* < 0.001) higher (Figure 2A) when volume and percentage of high intensity training (zone 3) increased initially at day 7 (23.24 ± 2.43 ng/ml), compared to day 0 (11.47 ± 3.92 ng/ml, *p* = 0.020) or to the even higher training load at day 13 (09.51 ± 3.59 ng/ml, *p* = 0.005) and subsequent time points with lower load (day 18: 10.79 ± 5.34 ng/ml, *p* = 0.014, and tapering phases day 24: 7.02 ± 1.75 ng/ml, *p* = 0.001 and day 28: 7.49 ± 1.80 ng/ml, *p* = 0.001). Significant differences (*p* < 0.0001) were also calculated for serum ferritin, mirroring the hepcidin time course when comparing day 7 (90.37 ± 30.51 ng/ml) to day 0 (68.50 ± 18.07 ng/ml, *p* = 0.001), day 18 (66.86 ± 20.92 ng/ml, *p* = 0.0003), day 24 (72.75 ± 19.34 ng/ml, *P* = 0.0112), and day 28 (63.62 ± 20.50 ng/ml, *p* < 0.0001).

No significant differences between high and low load training phases were detected for transferrin or iron (Figures 2B–D), albeit iron was significantly different between day 0 and day 24 (*p* = 0.028). A significant but weak positive correlation (*r* = 0.125, *p* = 0.022) was found between serum hepcidin and serum ferritin.

### No Significant Changes Were Found for the Ferritin Index and Expression of the Soluble Transferrin Receptor in Response to Increases in Training Load

**FIGURE 3:**
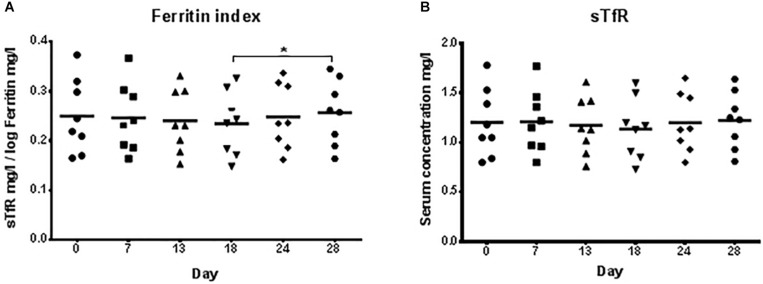
Ferritin index **(A)** and serum concentrations of the soluble transferrin receptor, sTfR **(B)** during low training load (day 0), high training load (day 7 and day 13), and moderate load before day 18, 24, and 28. ^∗^*p* < 0.05 day 18 vs. day 28.

There were no significant changes for the ferritin index (sTfR/log ferritin) and serum concentrations of sTfR (Figures 3A,B) between high and low load training phases indicating that there was no functional ID present in any of the athletes.

### High Training Load Does Not Alter Transferrin Saturation

**FIGURE 4:**
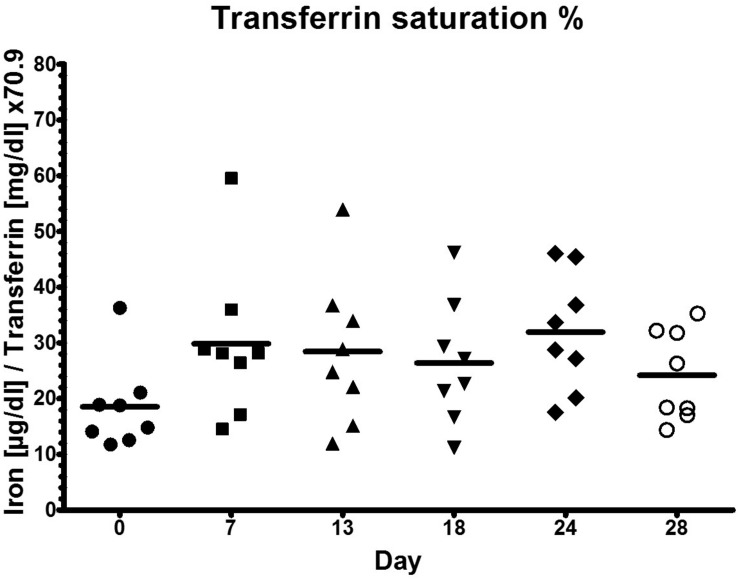
Transferrin saturation during low training load (day 0), high training load (day 7 and day 13), and moderate load before day 18, 24, and 28.

Since there were no significant changes for transferrin saturation (Figure 4) throughout the different phases of the training camp, there was no functional ID present in any athlete. Transferrin saturation was within the normal range >18–20% (Clénin et al., 2015).

### Hematocrit

Hematocrit, being a marker of longitudinal plasma volume alterations, changed not significantly (*p* = 0.292) within the training camp from day 0 to day 28 (48 ± 4%, 50 ± 3%, 50 ± 3%, 49 ± 4%, 53 ± 4%, 51 ± 3%).

## Discussion

World class rowers are regularly exposed to high workloads throughout the season and at risk of developing ID with or without anemia, due to reduced intestinal iron absorption, hemolysis, hematuria, sweating, and gastrointestinal bleeding resulting in compromised aerobic performance (Haas and Brownlie, 2001; Dellavalle and Haas, 2012). In addition, the hepatic acute-phase protein hepcidin contributes to impairments of iron status and exercise-induced anemia in endurance athletes due to iron compartmentalization resulting in functional ID (Peeling, 2010; Kong et al., 2014). Although the cut-offs for diagnosing functional ID vary, consensus suggests that diagnosis should include serum ferritin alongside of transferrin saturation (Anker et al., 2009; Eisenga et al., 2016). In athletic populations, a ferritin cut-off of 30 μg/l has been suggested (Clénin et al., 2015). Also, circulating concentrations of the sTfR and the ratio of sTfR to serum ferritin (sTfR-F index) have been used as sensitive and reliable indicators for functional ID (Malczewska et al., 2000, 2004; Schumacher et al., 2002b, c; Beguin, 2003; Nikolaidis et al., 2003).

The purpose of this study was to investigate circulating hepcidin levels in phases of different training loads in male junior world elite rowers and to relate its changes to indicators of iron status. Different to our hypothesis, serum iron levels as well as indicators of iron status (transferrin, sTfR) did not significantly change during the different training phases, despite significant differences in hepcidin and serum ferritin concentrations. However, the 3-fold increase of training load before day 7 was associated with significantly increased serum hepcidin levels and a tendency toward increased ferritin levels (Figures 2A,B). Interestingly, hepcidin levels returned to baseline levels at day 13, even though training load and volume increased further (Figure 1), indicating that the rowers were able to rapidly adapt to the high training load. Hematocrit (being the ratio of solid to fluid blood compartments) did not change significantly and was especially not elevated at day 7, indicating that the increase in hepcidin concentration was an absolute increase and not a dilution effect due to a dehydration associated decrease in plasma volume. Circulating serum iron and transferrin concentrations throughout the training camp remained in the normal ranges for young males of 7.2–27.7 μ mol/l and 2–4 g/l, respectively, demonstrating that iron was sufficiently available for erythropoiesis (Figures 2C,D). Since the ferritin index (sTfR/log ferritin) as well as serum concentrations of the sTfR (Figures 3A,B) were not significantly altered in response to high vs. lower load endurance training, and transferrin saturation levels were consistently >18% (Figure 4), we furthermore conclude that erythropoiesis was unaffected by iron compartmentalization through hepcidin and no functional ID was present in the male athletes at any time point.

A number of studies have shown that circulating hepcidin levels are transiently upregulated immediately after acute endurance exercise (Robson-Ansley et al., 2011; Antosiewicz et al., 2013; Skarpanska-Stejnborn et al., 2015; Tomczyk et al., 2017) and during the early (3 h) and late (5–6 h) recovery periods (Newlin et al., 2012; Sim et al., 2012, 2013, 2015; Badenhorst et al., 2015, 2016; Diaz et al., 2015; Dahlquist et al., 2017; Govus et al., 2017). For example, in Polish elite rowers, serum hepcidin, iron and IL-6 levels transiently increased immediately after an exhausting 2000 m rowing ergometer test with complete recovery after 24 h (Skarpanska-Stejnborn et al., 2015). These findings suggest that high intensity acute exercise is associated with a transiently increased hepcidin expression. In contrast, chronic endurance exercise training for 22 days in non-anemic, iron-sufficient male recreational athletes has been shown to reduce hepcidin levels while at the same time erythropoiesis was increased (Moretti et al., 2018). Also in rats, moderate intensity exercise has been shown to decrease rather than increase liver hepcidin mRNA expression after a 10-week swimming training (Liu et al., 2006). Most studies performed thus far have shown that hepcidin levels returned to baseline >6 h after acute endurance exercise (Newlin et al., 2012; Sim et al., 2012; Diaz et al., 2015; Skarpanska-Stejnborn et al., 2015). Our data show that resting serum hepcidin concentrations (12–15 h after the last training session) were elevated at the beginning of the high load training phase with a rapid return to baseline within only a few days, when rowers adapted to the high workloads (Figure 2A). Our findings are consistent with results from Ishibashi et al. (2017) who observed elevated hepcidin expression levels during an intensified training period in long-distance, female runners. Ma et al. (2013) compared resting serum hepcidin levels in highly trained female distance runners mid-season to a low exercise control group and showed that there was no difference between runners and controls for serum hepcidin levels mid-season, suggesting that hepcidin was not chronically elevated with sustained high training loads.

Hepcidin is regulated by iron status (Peeling et al., 2014; Burden et al., 2015), erythropoiesis (Pasricha et al., 2016), hypoxia (Liu et al., 2012; Govus et al., 2017), and inflammation (Nicolas et al., 2002), activating one of three main molecular pathways, including the JAK/STAT3, BMP/SMAD, and HFE/TfR2 pathways (Kong et al., 2014; Ruchala and Nemeth, 2014). High levels of plasma and liver iron have been shown to increase hepcidin transcription via the BMP pathway (Ramos et al., 2011), however, it remains to be further elucidated how iron is exactly sensed intracellularly. The elevated hepcidin levels during the initial increase in training load in this study cannot be explained by changes in iron loading, since circulating iron and ferritin levels were not significantly elevated at any time point. Further speculative reasons for the hepcidin increase may be a decreased clearance of hepcidin in its steady state, reduced levels of negative regulators of hepcidin, such as anti-inflammatory cytokines, erythropoiesis-stimulating agents and increased levels of pro-inflammatory cytokines, or even an exercise induced hepatic hypoperfusion (Rowell et al., 1968), which is triggered by sympathetic nervous system activation (Nielsen, 2003).

Hepcidin expression levels are rapidly increased by inflammation. The regulation of hepcidin by inflammatory signals has evolved evolutionary as a host defense mechanism to reduce iron availability for iron-dependent pathogens (Ruchala and Nemeth, 2014). Furthermore, it is conceivable that exercise-induced increases in hepcidin serve as an early mechanism to avoid excessive circulating iron in addition to toxic iron release by exercise-induced necrotic cells. Our own data and others have described exhaustive exercise as a source of cell free DNA (Velders et al., 2014) as well as HMGB1 (Scaffidi et al., 2002) being indicators for dying cells and the uncontrolled release of their intracellular contents. There are reports indicating that increases in serum hepcidin levels in the hours post-exercise are preceded by an increase in the expression of the myokine IL-6, which induces hepcidin expression through the JAK/STAT3 signaling pathway (Wrighting and Andrews, 2006; Peeling et al., 2009; Robson-Ansley et al., 2011; Banzet et al., 2012). However, hepcidin levels were unchanged after a 100 km run despite an increase in inflammatory markers, suggesting that high training volume without sufficient high intensity (i.e., zone 3-training) components is insufficient to raise hepcidin levels (Kasprowicz et al., 2013). Peeling et al. (2014) showed that the exercise-induced increase in hepcidin was suppressed in athletes with low pre-exercise iron stores (serum ferritin levels <30 μg/l) compared to those with higher iron stores, despite similar post-exercise increases in the myogenesis factor IL-6 with its special metabolic function at conditions of physical exhaustion. Similarly, iron supplementation in iron deficient, non-anemic endurance athletes increased post-exercise hepcidin expression levels compared to placebo, independent of changes in IL-6 (Burden et al., 2015). Also, an iron deficit has been shown to blunt the hepcidin response to an inflammatory stimulus such as lipopolysaccharide (LPS) (Darshan et al., 2010). Taken together, findings suggest that the pre-exercise iron status may be a more powerful regulator of hepcidin expression compared to sterile inflammation.

The circulating sTfR concentration is proportional to cellular expression of the membrane-associated TfR and increases with amplified cellular iron needs and cellular proliferation (R’Zik and Beguin, 2001). Circulating sTfR levels and the sTfR/log ferritin index have been shown to be stable under high physical training loads. While acute, intense exercise was associated with temporarily elevated sTfR levels, which can be attributed to changes in plasma volume and the resulting increase in hemoconcentration during and immediately after acute exercise, exercise training has none or minimal effects on sTfR levels (Malczewska et al., 2000, 2004; Schumacher et al., 2002c). The ferritin index has a lower mean day-to-day variability (11.8%, range: 5–21%) compared to ferritin (27.4%, range: 16–44%) (Malczewska et al., 2004) and remained stable despite day to day changes in ferritin and sTfR levels (Auersperger et al., 2013). Therefore, sTfR concentration and the sTfR/log ferritin index are presumably the most reliable markers to assess iron status and erythropoiesis in athletes. Furthermore, because serum ferritin reflects the storage iron compartment and sTfR reflects the functional iron compartment, the sTfR/log ferritin index has been suggested as a good estimate of total body iron (Kotisaari et al., 2003). Our results show that transferrin saturation remained stable without significant changes throughout the study period and was within the normal healthy range of 18–45%. Similarly, the ferritin index (sTfR/log ferritin) was <1.5 and changed not significantly during the training phases, suggesting that there were no deficits in whole body iron present in the athletes. Therefore, iron metabolism in highly trained endurance athletes may be unaffected by short-term oscillations in hepcidin expression. In line with this, McClung et al. (2013) showed that chronic exercise (7-day military training march) resulted in significant elevations of IL-6 and serum hepcidin levels without significant changes in sTfR. Ferritin and transferrin levels have been shown to increase in response to acute exercise, most likely due to higher hemoconcentrations (Schumacher et al., 2002b). Ferritin levels were increased after prolonged endurance exercise in elite male cyclists, while transferrin remained unchanged (Schumacher et al., 2002c). We confirm these results by showing that transferrin levels were unaffected by the differences in training load, while ferritin levels were significantly increased in response to the increase in training load at the beginning of the training camp (day 7), indicating an acute phase response of ferritin and hepcidin in concert.

Results from Reinke et al. (2012) showed that at the end of a competitive season, 27% of senior elite rowers and professional soccer players were diagnosed with absolute ID and 70% with functional ID based on the clinical definition of functional ID (ferritin 30–99 μg/l or 100–299 μg/l + transferrin saturation <20%) (Anker et al., 2009; Reinke et al., 2012; Eisenga et al., 2016). However, a ferritin cut-off of 30 μg/l has been suggested for adult athletes in a consensus statement of the Swiss Society of Sports Medicine (Clénin et al., 2015). According to this definition and the normal range values of iron, transferrin, sTfR, ferritin index, and transferrin saturation, we rather conclude that none of the elite rowers in this study suffered from absolute or functional ID. The highly trained endurance athletes in our study were able to compensate and adapt to the cumulative training stimuli during the training camp. This conclusion is furthermore underlined by the excellent competition outcome of the participants (Table 1).

Important and noteworthy, based on these data and the fact that iron and hepcidin contribute to iron homeostasis in a feedback system, it is conceivable that excessive iron supplementation may lead to further hepcidin-induced iron retention in the mononuclear phagocyte system and functional ID, inducing decrements in endurance performance. Interestingly, in a study of Mettler et al., among 127 male recreational marathon runners only <2% suffered from iron depletion while 9-fold more athletes were diagnosed with iron overload presumably in an attempt to increase performance (Mettler and Zimmermann, 2010). Ishibashi et al. (2017) showed that serum hepcidin levels were higher in female runners with iron supplementation. Also, iron supplementation can increase serum ferritin without increasing hemoglobin concentrations (Garza et al., 1997). Accordingly, a single intravenous iron injection (500 mg) in iron-deficient runners increased serum iron, hepcidin and ferritin expression post-exercise for at least four weeks without improving aerobic capacity (Burden et al., 2015), and underlines once more the double-edged sword of dietary supplementation without an acute clinical indication (Maughan et al., 2018).

The results of our study are limited by the small sample size, being a frequent limitation of high-performance sport studies, the lack of inflammatory data in addition to ferritin, and finally the lack of hematological data, especially reticulocyte count and hemoglobin mass.

In summary, our findings indicate that hepcidin as well as ferritin representing acute phase proteins, are sensitive to initial increases in training load (i.e., increases in volume and percentage of high intensity training) in male junior world elite rowers without causing short-term alterations in functional iron homeostasis. Unaltered sTfR levels and the lack of changes in the ferritin index indicate that erythropoiesis was unaffected by iron compartmentalization through hepcidin. We like to emphasize though that our findings should not be generalized and might be different in other populations like e.g., menstruating female rowers, especially with hypermenorrhea. Also, insufficient nutritional iron availability might be a modulating factor for individual hepcidin response to training load. In future studies, additional markers of erythropoiesis should be monitored, such as the newly identified hormone erythroferrone, mediating hepcidin suppression during stress erythropoiesis (Kautz et al., 2014; Pasricha et al., 2016), the reticulocyte hemoglobin (RET-Hb) content, and the percentage of hypochromic red blood cells (Hypo%), being present when hemoglobin concentrations are <280 g/l (Buttarello et al., 2010; Eguchi et al., 2012). In combination with markers of inflammation (e.g., hsCRP, TNF-α) during various training and competition periods, a longitudinal and individual iron-status profile might help to prevent functional ID in elite endurance athletes. Finally it would be enlightening to gather more information on iron status in overtrained athletes, representing a phenotype with declines in performance, and to compare the respective molecular patterns between women and men.

## Data Availability Statement

The datasets generated for this study are available on request to the corresponding author.

## Ethics Statement

The ethical review board of the University of Ulm approved this study (121-09) which complied with the Declaration of Helsinki. All participants gave written informed consent and parental consent was obtained for athletes <18 years of age.

## Author Contributions

MZ, GT, JS, and US designed the study. GT and JS collected the samples. MZ and US analyzed the samples. MZ, GT, BM, and US analyzed the data. MZ, GT, and US drafted the manuscript. MZ, GT, JS, BM, KW, and US edited the manuscript.

## Conflict of Interest

The authors declare that the research was conducted in the absence of any commercial or financial relationships that could be construed as a potential conflict of interest.
